# Impacto da COVID-19 nos Programas de Reabilitação Cardiovascular no Brasil: Um Estudo Transversal Baseado em uma Pesquisa Online

**DOI:** 10.36660/abc.20220135

**Published:** 2023-02-23

**Authors:** Iara de Sousa Cezário Jardim, Mauricio Milani, Isac Castro, Dominique Hansen, Marlus Karsten, Lawrence Patrick Cahalin, Graziella França Bernardelli Cipriano, Gerson Cipriano

**Affiliations:** 1 PPGCR Universidade de Brasília Brasília DF Brasil Programa de Pós Graduação em Ciências da Reabilitação (PPGCR), Universidade de Brasília (UNB), Brasília, DF – Brasil; 2 Fitcordis Brasília DF Brasil Fitcordis, Brasília, DF – Brasil; 3 PPGCTS Universidade de Brasília Brasília DF Brasil Programa de Pós Graduação em Ciências e Tecnologias em Saúde (PPGCTS), Universidade de Brasília (UNB), Brasília, DF – Brasil; 4 Universidade de São Paulo São Paulo SP Brasil Universidade de São Paulo, São Paulo, SP – Brasil; 5 Hasselt University Hasselt Bélgica Hasselt University, Hasselt – Bélgica; 6 PPGFT Universidade do Estado de Santa Catarina Florianópolis SC Brasil Programa de Pós-graduação em Fisioterapia (PPGFT), Universidade do Estado de Santa Catarina, Florianópolis, SC – Brasil; 7 Miller School of Medicine Miami Flórida EUA Miller School of Medicine, Miami, Flórida – EUA

**Keywords:** COVID-19, Telerreabilitação, Reabilitação Cardíaca, Pesquisa, Inquéritos e Questionários

## Abstract

**Fundamento:**

A pandemia da COVID-19 teve um impacto sobre os programas de reabilitação cardiovasculares (RC) no Brasil.

**Objetivos:**

Descrever características dos programas de RC no Brasil, os impactos da primeira onda epidemiológica da COVID-19 (primeiros 60 dias) sobre os programas, e apresentar as iniciativas usadas para superar esses impactos.

**Métodos:**

Este estudo transversal e retrospectivo usou um questionário online específico. Os participantes eram coordenadores de programas de RC. As variáveis foram apresentadas por região geográfica do Brasil, como as seguintes categorias: característica demográficas, clínicas e operacionais. O nível de significância estatística foi definido em 5%.

**Resultados:**

Cinquenta e nove programas de RC atendiam 5349 pacientes, dos quais somente 1817 eram pacientes após eventos cardiovasculares agudos, o que correspondia a 1,99% dos pacientes internados no mês anterior à pesquisa (n=91.231). O maior impacto foi a suspensão das atividades presenciais, o que ocorreu de maneira similar em áreas com as taxas mais altas e áreas com as taxas mais baixas de COVID-19 no período. Quarenta e quatro (75%) programas foram interrompidos de forma breve, e três (5%) foram encerrados. Todos os 42 programas que já utilizavam estratégias de reabilitação remota notaram aumento substancial nas atividades, baseadas principalmente no uso da mídia e chamadas por vídeo. Somente três (5%) consideraram seguro atender pacientes durante os primeiros 60 dias.

**Conclusões:**

Houve redução no número de programas de RC devido a pandemia da COVID-19. Atividades de telerreabilitação aumentaram durante os primeiros dois meses da pandemia da COVID-19, mas que não foi suficiente para superar a redução nas atividades dos programas de RC no Brasil.

## Introdução

Em março de 2020, uma pandemia mundial foi declarada devido à emergência da COVID-19 ( *coronavirus disease* 2019).^1^ Não havia nenhum medicamento específico ou vacinas regulamentadas para tratar a doença causada pelo SARS-CoV-2 durante a primeira metade de 2020.^2^ Medidas preventivas para reduzir a rápida disseminação desse vírus respiratório, incluindo distanciamento social, foram então recomendadas em todo o mundo.^3^ A recomendação de se reduzir o contato físico, feita por agências de saúde, aumentou as restrições no acesso a muitas áreas públicas e privadas, incluindo a serviços de saúde.^4^ Esse fenômeno também ocorreu no Brasil, onde o primeiro caso relatado foi em 25 de fevereiro de 2020, nove semanas após o primeiro caso relatado na China.^5^ Em Brasília, por exemplo, o governo lançou o primeiro decreto para o fechamento de vários estabelecimentos em 11 de março de 2020 (11ª semana epidemiológica).^6^ Tais recomendações continuaram a mudar ao longo do ano, e alterações nas restrições ainda eram anunciadas nos primeiros meses de 2021.^7^

De acordo com publicações recentes, condições crônicas, incluindo doenças cardiovasculares e doenças pulmonares, diabetes mellitus, e hipertensão, foram associadas a manifestações mais graves da infecção por COVID-19 e pior prognóstico.^8 , 9^ Assim, indivíduos com essas condições foram solicitados a aumentar as medidas de precaução e manter os tratamentos clínicos e, assim, evitar contaminação, descompensação e outras internações.^10^

Embora considerada um componente vital no tratamento da insuficiência cardíaca,^11 , 12^ a disponibilidade e a adesão à reabilitação cardiovascular (RC) também foram também foram muito afetadas devido às novas restrições durante a pandemia.^13^ A RC é composta de componentes chave específicos que têm como objetivo estimular hábitos saudáveis, promover um estilo de vida ativo, otimizar condições clínicas, e reduzir riscos cardiovasculares,^11^ corroborando evidências robustas de sua capacidade em prevenir hospitalizações e melhorar a aptidão cardiorrespiratória de maneira custo-efetiva.^14 , 15^

Este estudo tem como objetivo descrever as características dos Programas de Reabilitação Cardíaca (PRCs) no Brasil antes e durante a pandemia, verificar o impacto da pandemia da COVID-19 (60 dias após o primeiro caso confirmado da COVID-19 no Brasil), e descrever as principais iniciativas criadas para manter os PRCs face às restrições impostas por autoridades públicas.

## Métodos

### Aprovação ética e delineamento metodológico

Neste estudo retrospectivo, transversal, utilizou-se uma pesquisa *online* desenvolvida por profissionais de RC e pesquisadores. O estudo foi aprovado pelo comitê de ética (número CAAE 36041220.4.0000.8093). Os participantes assinaram um acordo de confidencialidade descrevendo o propósito científico e o anonimato na apresentação de seus dados durante um webinar científico internacional, organizado pelo *International Council of Cardiovascular Prevention and Rehabilitation* .^16^

### Pesquisa *online*

A pesquisa *online* foi elaborada por dois pesquisadores, para apresentar, pela primeira vez na literatura, dados brasileiros sobre o impacto da COVID-19 na RC.^16^ Os dados completos coletados foram registrados para posterior avaliação após a aprovação ética.

Um programa gratuito (Google forms, Google LLC, California, Estados Unidos) foi usado para desenvolver questões objetivas e subjetivas do questionário. O questionário foi distribuído entre 20 e 30 de abril de 2020 (60 dias após o primeiro caso confirmado de COVID-19 no Brasil). A sequência das questões teve como objetivo primário caracterizar os serviços de RC antes da pandemia da COVID-19 e, segundo, apresentar adaptações potenciais para o novo cenário da pandemia. Embora não houvesse limite de tempo para os participantes completarem o questionário, o tempo aproximado para o preenchimento do formulário foi de 10 a 15 minutos por participante. Cada página tinha de ser completamente respondida para o total preenchimento do questionário. A versão em português usada no estudo ainda está disponível *online* para consulta (https://forms.gle/hWiKojuAz68FpUeD6)

Os profissionais da RC também foram solicitados a concordar com os termos de divulgação, consentindo com a apresentação anônima dos dados no *webinar* da ICCPR e publicação científica após aprovação pelo comitê de ética.

### Participantes, e critérios de inclusão e exclusão

Os participantes do estudo eram coordenadores de PRCs selecionados por amostragem não probabilística. O *link* para acessar o questionário *online* foi disponibilizado por redes sociais, e os receptores foram encorajados a enviar o convite a outros profissionais para alcançar o maior número possível de profissionais (isto é, estratégia “bola de neve”).

Como critérios de inclusão, o PCR tinha que oferecer ao menos um dos oito componentes centrais recomendados na literatura.^11^ Questionários em duplicata ou respondidos apenas parcialmente foram excluídos do estudo.

### Análise estatística

O teste de Kolmogorov-Smirnov foi usado para verificar a normalidade da distribuição dos dados da amostra. Como os dados não apresentaram distribuição normal, as variáveis contínuas foram descritas em mediana e intervalo interquartil. Os dados categóricos foram apresentados como frequência absoluta e porcentagens. Os dados foram analisados usando o teste de McNemar, em uma tabela 2x2, considerando: 1) a estratégia promoveu mudança de comportamento; 2) começou a usar estratégia ou interrompeu seu uso em dois momentos – antes e após a pandemia. Os dados categóricos foram comparados usando o teste do X^2^. O teste de Kruskal-Wallis foi usado para comparar medianas de dados não paramétricos. Usamos um p<0,05 e um erro beta ≤ 0,2 para significância estatística. As análises estatísticas foram realizadas usando o programa SPSS, versão 23.0 para MacOS.

Um total de 57 PRCs seria necessário (76% dos participantes). Esse número foi calculado usando um z-escore de 1,96 e margem de erro de 6%, e considerando um número total estimado de 75 PRCs no Brasil.^17^ Os cálculos foram realizados usando o programa StatCatl, EPIinfo, e os questionários populacionais do CDC ( *Centers for Disease Control and Prevention* ). Para estimar a demanda por PRC no Brasil e por região geográfica, consideramos o número de internações por doença cardiovascular no mês anterior à pesquisa – março de 2020,^18^ (uma vez que o número de internações foi similar à média de 12 meses anteriores), dividido pelo número de sessões de RC potencialmente disponível para os pacientes que tiveram um evento cardiovascular agudo recente (<12 semanas).

Para calcular a taxa média de infecção por COVID-19 no mês de abril de 2020 por região geográfica, nós consideramos o número de casos registrados no sistema nacional de saúde em 2020,^19^ dividido pelo número de habitantes registrados pelo Instituto Brasileiro de Geografia e Estatística para o mesmo período.^20^ A razão foi expressa por 100 000 habitantes.

## Resultados

### Características dos programas de reabilitação cardiovascular antes da pandemia da COVID-19

A taxa de resposta ao nosso questionário foi de 78,66% do número esperado (61 de 75 programas). Dois programas foram excluídos por duplicidade, resultando em 59 questionários para análise.

As características dos PRCs antes da pandemia da COVID-19 foram categorizadas de acordo com as regiões geográficas do Brasil – norte (N), nordeste (NE), centro-oeste (CO), sudeste (SE), e sul (S) ( Tabela 1 ).

**Tabela 1:** – Características clínicas e demográficas dos programas de reabilitação cardiovascular antes do primeiro caso de COVID-19 confirmado no Brasil e internações por doenças cardiovasculares por região brasileira

Características demográficas		Região brasileira					Total	p

		Centro-oeste	Nordeste	Sul	Sudeste	Norte		
Perfil do serviço oferecido em mediana (IIQ) ou *n (%)*	Tempo de existência (meses)	77 (42 -131)	68 (28 - 96)	57 (20 - 229)	141 (70 - 238)	-	-	0,26
	Número de programas	9 (15,25)	8 (13,55)	12 (20,33)	30 (50,84)	-	59	-
	Índice de desenvolvimento humano	0,757	0,663	0,754	0,766	0,667	-	-
	Número de pacientes por mês (por programa)	50 (20 - 50)	18 (7 - 65)	25 (7 - 115)	78 (49 - 180)	-	5.349	0,01
	Número de pacientes por mês	428 (8)	270 (5,04)	985 (18,41)	3,711 (69,37)	-		
	Número de sessões por mês (por programa)	200 (12 - 280)	76 (12 - 220)	40 (10 - 132)	180 (50 - 400)	-	32.449	0,25
	Número de sessões por mês	3.016 (9,29)	1.764 (5,43)	5.080 (15,65)	22.589 (69,61)	-		
	Porcentagem de pacientes em baixo risco* por mês (por programa)	30 (20 - 50)	15 (5 - 55)	30 (10 - 50)	25 (20 - 50)	-	1.407	0,54
	Porcentagem de pacientes em risco moderado* por mês (por programa)	50 (30 - 60)	50 (20 - 65)	35 (20 - 50)	35(20 - 5)	-	2.145	0,27
	Porcentagem de pacientes em alto risco* por mês (por programa)	20 (10 - 20)	30 (10 - 50)	25 (5 - 55)	30 (20 - 50)	-	2.097	0,31
	Porcentagem de pacientes após evento cardiovascular agudo (<12 semanas) por mês (por programa)	20 (20 - 30)	15 (0 - 25)	10 (5 - 30)	20 (20 - 30)	-	1.817	0,30
	Número de pacientes após evento cardiovascular agudo (<12 semanas) por mês (por programa)	220 (12,1)	557 (30,7)	230 (12,7)	810 (44,6)	-		
	Pacientes do PRC usuários do sistema público de saúde (por programa)	0 (0 - 0)	5 (0 - 45)	25 (0 - 25)	70 (0 - 70)	-	1.173	0,48
Hospitalizações por DCV em março de 2020^29^		6.778 (7,4)	20.127 (22)	20.395 (22,3)	39.573 (43,3)	4.358 (4,7)	91.231	-
Pacientes após evento agudo/pacientes internados por DCV em março de 2020 (%)		3,24	2,76	1,12	2,04	0	1,99	-
**Características clínicas**		**Região brasileira**					**Total**	**p**

		**Centro-oeste**	**Nordeste**	**Sul**	**Sudeste**	**Norte**		
Número (%) de programas direcionados a condições específicas	Insuficiência cardíaca	8 (88,9)	8 (100)	9 (75)	30 (100)	0	55 (98)	0,02
	Revascularização coronária	8 (88,9)	7 (87,5)	9 (75)	29 (96,7)	0	53 (89)	0,21
	Após implante de marca-passo e/ou CDI	6 (66,7)	6 (75)	7 (58,3)	25 (83,3)	0	44 (74)	0,36
	Doença coronariana estável	6 (66,7)	5 (62,5)	6 (500	25 (83,3)	0	42 (71)	0,16
	Síndrome coronariana aguda	7 (77,8)	5 (62,5)	7 (58,3)	22 (73,3)	0	41 (69)	0,70
	Síndrome metabólica	7 (77,8)	5 (62,5)	6 (50)	23 (76,7)	0	41 (69)	0,34
	Diabetes	6 (66,7)	4 (50)	6 (50)	23 (76,7)	0	39 (66)	0,28
	Pós-cirurgia valvar	5 (55,6)	4 (50)	5 (41,7)	21 (70)	0	35 (59)	0,34
	DAOP	2 (22,2)	3 (37,5)	6 (50)	18 (60)	0	30 (50)	0,21
	Cardiopatias congênitas no adulto	3 (33,3)	2 (25)	0 (0)	11 (36,7)	0	16 (27)	0,11
	Pós-transplante cardíaco ou DAV	0 (0)	2 (25)	1 (8,3)	13 (43,3)	0	16 (27)	0,02
	Outros	1 (11,1)	3 (37,5)	2 (16,7)	7 (23,3)	0	13 (22)	0,51
	Pós-evento cerebrovascular	1 (11,1)	1 (12,5)	1 (8,3)	9 (30)	0	12 (20)	0,31
	Pré-operatório de cirurgia cardíaca	0 (0)	0(0)	1 (8,3)	2 (6,7)	0	3 (5)	0,72

Dados expressos em número absoluto (n) e porcentagem (%) para variáveis categóricas e em mediana e intervalo interquartil (25-75%) para variáveis contínuas. A comparação entre grupos foi analisada por X^*2*^ de Pearson (p< 0,05). IIQ: intervalo interquartil; N: número; *: risco baixo, moderado ou alto, segundo diretriz Brasileira^*26*^ ; PRC: Programa de reabilitação cardíaca; DCV: doença cardiovascular; p-valor: teste qui-quadrado de Pearson; CDI: cardiodesfibrilador implantável; DAOP: doença arterial obstrutiva periférica; DAV: dispositivo de assistência ventricular.

**Figura 1:**
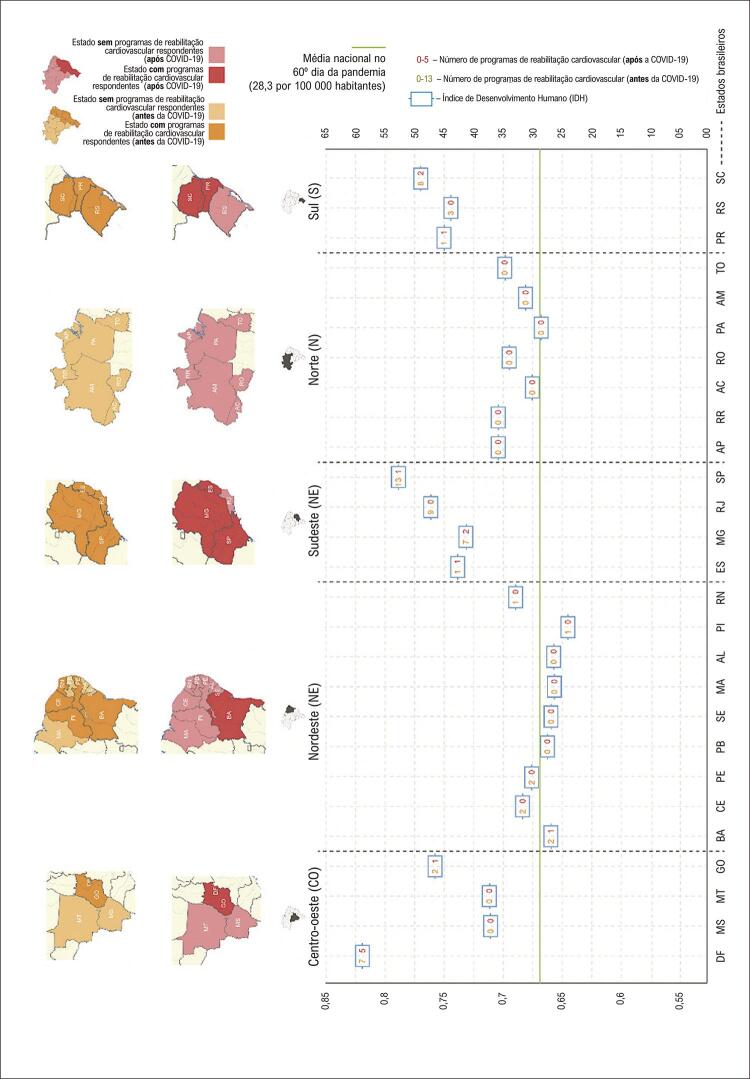
– Número de programas de reabilitação cardiovascular e taxa de infecção pelo novo coronavírus (COVID-19) antes e após a primeira onda da doença (20 a 30 de abril de 2020), e Índice de Desenvolvimento Humano (IDH) por unidade federativa e região do Brasil.

A maioria dos PRCs participantes do estudo (n=44, 74%) estavam localizados em quatro estados brasileiros, os quais apresentavam o maior Índice de Desenvolvimento Humano (IDH) (acima de 0,731), de acordo com a literatura.^21^ Considerando a taxa média de infecção por COVID-19 para o período avaliado (28,3 por 100 000 habitantes), três desses quatro estados estavam acima do número calculado ( Figura 1 ). Segundo dados disponíveis na literatura,^21^ a região N apresentava o segundo pior IDH no Brasil (0,667) e foi a única região em que não houve nenhum respondente ao questionário. Por outro lado, a região SE que tem o maior IDH do país (0,766),^21^ a maior concentração de PRCs (30/59), o programa mais antigo (238 meses), a maior taxa de pacientes tratados por programa (78%), e o maior número de pacientes assistidos pelo sistema público de saúde (SUS) (70%).

De acordo com o número de internações por doenças cardiovasculares no Brasil em março de 2020 (n=91.231), e a disponibilidade de programas para pacientes após os eventos cardiovasculares agudos (n=1.817), somente 1,99% dos pacientes puderam ser tratados em PRCs. O pior cenário ocorreu na região S (número de programas/número de pacientes: 230/20.395: 1,12%), seguido da região SE (810/39.573: 2,04%) ( Tabela 1 ).

Quanto às características clínicas, a insuficiência cardíaca (IC) foi a condição clínica mais descrita pelos coordenadores de PRCs (n=55, 93,2%), seguido de revascularização coronariana (n=53, 89,8%) e pacientes com marca-passo ou desfibrilador cardíaco implantável (n=44, 74,5%) ( Tabela 1 ). Contudo, a porcentagem de programas direcionados a pacientes com IC foi diferente entre as regiões geográficas do Brasil. Enquanto 100% dos PRCs nas regiões NE e SE atendiam pacientes com IC, somente 89% e 75% dos programas nas regiões CO e S, respectivamente, eram direcionados a esses pacientes (p=0,02) ( Tabela 1 ). Nenhum dos programas da região CO atendia pacientes no pós-transplante cardíaco e/ou usuários de suporte ventricular; por outro lado, 43,3% dos PRCs na região SE atendia pacientes no pós-transplante (p=0,02) ( Tabela 1 ).

Encontramos discrepâncias importantes quanto aos componentes chave empregados por PRCs entre as regiões. Aconselhamento nutricional foi oferecido em um programa (11%) na região CO, e em 19 programas (63,3%) na região SE (p=0,005) ( Tabela 2 ). Suporte vocacional foi o componente menos oferecido no Brasil (n= 1, 8,3%), ao passo que orientação sobre exercício (n=59, 100%) e atividade física (n=57, 96%) foram os componentes mais frequentes ( Tabela 2 ).

**Tabela 2:** – Características operacionais dos programas de reabilitação cardíaca antes do primeiro caso de COVID-19 confirmado no Brasil e internações por doenças cardiovasculares por região brasileira

Características operacionais		Região brasileira						Total	p

		Centro-oeste	Nordeste	Sul	Sudeste		Norte		
Número (%) de programas que ofereceram componentes centrais	Exercícios	9 (100)	8 (100)	30 (100)		12 (100)	0	59 (100)	1
	Aconselhamento nutricional	1 (11,1)	6 (75)	10 (83,3)		19 (63,3)	0	36 (61)	0,005
	Controle de fatores de risco	7 (77,8)	7 (87,5)	9 (75)		26 (86,7)	0	49 (83)	0,07
	Cessação do tabagismo	1 (11,1)	2 (25)	3 (25)		9 (30)	0	15 (25)	0,72
	Suporte psicológico	1 (11,1)	3 (37,5)	5 (41,7)		12 (40)	0	21(35)	0,42
	Orientação sobre atividade física	8 (88,9)	8 (100)	11(91,7)		30 (100)	0	57 (96)	0,27
	Orientação vocacional	0 (0)	0 (0)	1 (8,3)		0 (0)	0	1 (1)	0,80
	Controle de adesão ao tratamento	7 (77,8)	7 (87,5)	8 (66,7)		25 (83,3)	0	47 (79)	0,61
Métodos de avaliação usados pelos programas *n (%)*	Teste cardiopulmonar de exercício	4 (44,4)	7 (87,5)	6 (50)		20 (66,7)	0	37 (62)	0,22
	Teste de caminhada de 6 minutos	7 (77,8)	4 (50)	11(91,7)		17 (56,7)	0	39 (66)	0,10
	Teste de levantar e sentar	3 (33,3)	4 (50)	7 (58,3)		12 (40)	0	26 (44)	0,36
	Teste de preensão manual	2 (22,2)	3 (37,5)	6 (50)		9 (30)	0	20 (33)	0,53
	Força muscular respiratória	4 (44,4)	6 (75)	7 (58,3)		17 (56,7)	0	34 (57)	0,65
	Teste de flexibilidade	3 (33,3)	3 (37,5)	4 (33,3)		14 (46,7)	0	24 (40)	0,81
	Mobilidade funcional – teste *timed up and go*	2 (22,2)	1(12,52)	4 (33,3)		8 (26,7)	0	15 (25)	0,75
	Avaliação do equilíbrio – tese de Berg	1 (11,1)	2 (25)	1 (8,3)		9 (30)	0	13 (22)	0,37
	Avaliação da qualidade de vida	1 (11,1)	2 (25)	1 (8,3)		2 (6,7)	0	6 (10)	0,49
Tipos de treinamento oferecidos pelos programas *n (%)*	Treinamento de coordenação motora	4 (44,4)	5 (62,5)	6 (50)		25 (83,3)	0	40 (68)	0,06
	Treino de equilíbrio	5 (55,6)	6 (75)	8 (66,7)		27 (90)	0	46 (78)	0,10
	Alongamento	8 (88,9)	8 (100)	12 (100)		30 (100)	0	58 (98)	0,13
	Treinamento muscular respiratório de intensidade moderada	2 (22,2)	6 (75)	6 (50)		17 (56,7)	0	31 (53)	0,16
	Treinamento de resistência de intensidade moderada	7 (77,8)	7 (87,5)	11 (91,7)		28 (93,3)	0	53 (90)	0,58
	Treino intervalado de alta intensidade	5 (55,6)	6 (75)	4 (33,3)		17 (56,7)	0	32 (54)	0,31
	Treinamento contínuo de intensidade moderada	8 (88,9)	8 (100)	11 (91,7)		30 (100)	0	57 (97)	0,27
Estratégias usadas para oferecer reabilitação cardíaca remota *n (%)*	Chamadas telefônicas	2 (22,2)	3 (37,5)	4 (33,3)		11 (36,7)	0	20 (33)	0,87
	Aplicativo ou software para PRC	0 (0)	0 (0)	0 (0)		0 (0)	0	0 (0)	1,00
	Suporte de mídia (vídeos e fotos)	0 (0)	1 (12,5)	2 (16,7)		5 (16,7)	0	8 (13)	0,62
	Material promocional	1 (11,1)	1 (12,5)	2 (16,7)		11 (36,7)	0	16 (27)	0,24
	Chamadas por vídeo	0 (0)	1 (12,5)	2 (16,7)		4 (13,3)	0	7 (11)	0,67
	Outros	1 (11,1)	1 (12,5)	1 (8,3)		4 (13,3)	0	7 (11)	0,97

*Dados expressos em número absoluto (n) e porcentagem (%) para variáveis categóricas; comparações entre grupos foram analisadas pelo teste de Kruskal-Wallis (p<0,05); PRC: Programa de reabilitação cardiovascular; p-valor: teste de Kruskal-Wallis.*

As principais modalidades de exercícios presenciais oferecidas nos PRCs foram alongamento (98%), treinamento aeróbico contínuo de intensidade moderada (97%), e treinamento de resistência de intensidade moderada (90%) ( Tabela 2 ). Nenhum dos 59 programas utilizava software ou aplicativo específico de RC antes da pandemia da COVID-19; chamadas telefônicas (n=20, 33%) e materiais promocionais (n=16, 27%) eram as iniciativas remotas mais prevalentes antes da pandemia ( Tabela 2 ).

### Impacto da COVID-19 sobre programas de reabilitação cardiovascular no Brasil

De acordo com a média nacional de infecção por COVID-19, os estados brasileiros foram diferentemente afetados em abril de 2020 (28,3 por 100,000 habitantes). Na região SE, 23 (63,9%) dos PRCs ocorreram em cidades com uma taxa de infecção acima da média nacional, enquanto 5,6% dos PRCs na região S, 19,4% na região CO, e 11,1% na região NE foram conduzidos em cidades com uma taxa de infecção acima da média nacional (p=0,002) ( Tabela 3 ).

**Tabela 3:** – Local do programa de reabilitação cardiovascular de acordo com a taxa nacional média de COVID-19 19 (> ou ≤28,3 por 100000 habitantes) por região brasileira e os principais impactos dos programas nos primeiros 60 dias após o primeiro caso de COVID-19 no Brasil

Regiões brasileiras/ Impactos da pandemia	Taxa nacional média de COVID calculada nos primeiros 60 dias da pandemia							Teste do qui-quadrado de Pearson

	< = 28,3 por 100 000 habitantes			> 28,3 por 100 000 habitantes				

	n		%	n	%	Total (n)	Total (%)	
Centro-oeste	2	8,7		7	19,4	9	15,2	p = 0,002
Nordeste	4	17,4		4	11,1	8	13,6	
Sudeste	7	30,4		23	63,9	30	50,9	
Sul	10	43,5		2	5,6	12	20,3	
*Total*	*23*	*100*		*36*	*100*	59	100	
Encerramento do programa	2	8,7		1	2,8	3	5	p = 0,09
Interrupção do programa	15	65,2		30	83,3	45	76,3	
Redução no número de sessões	6	26,1		2	5,6	8	13,6	
Funcionamento normal do programa	0	0		1	2,8	1	1,7	
Outros	0	0		2	5,5	2	3,4	
***Total***	*23*	*100*		*36*	*100*	59	100	

*Variáveis categóricas expressas em número absoluto (n) e porcentagem (%). Comparação entre grupos analisada por teste qui-quadrado de Pearson (p< 0,05).*

O maior impacto da pandemia da COVID-19 sobre os PRCs foram as restrições comerciais na cidade, que ocorreram independentemente da taxa de infecção local, já que 30 dos 36 (83,3%) programas foram realizados em cidades com uma taxa acima da média, e 15 dos 23 (65,2%) programas eram em cidades com uma taxa abaixo da média ( Tabela 3 ).

Uma redução no número de sessões de RC foi o segundo maior impacto, observada em 26,1% das cidades com as maiores taxas de infecção por COVID-19, e em 5,6% das cidades com as taxas mais baixas de infecção por COVID-19 (p=0,097) ( Tabela 3 ).

**Figura 2:**
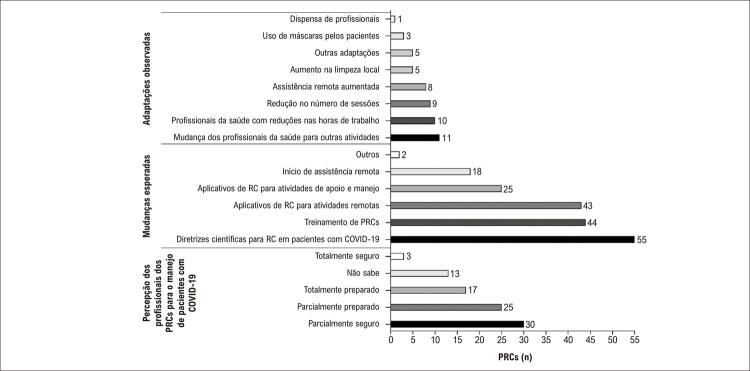
– Principais mudanças observadas e esperadas nos Programas de Reabilitação Cardiovascular (PRCs), impostas pela pandemia da COVID-19, e percepção dos profissionais dos PRCs sobre o manejo dos pacientes com COVID-19 durante a primeira onda da pandemia (20 a 30 de abril de 2020) no Brasil.

**Figura 3:**
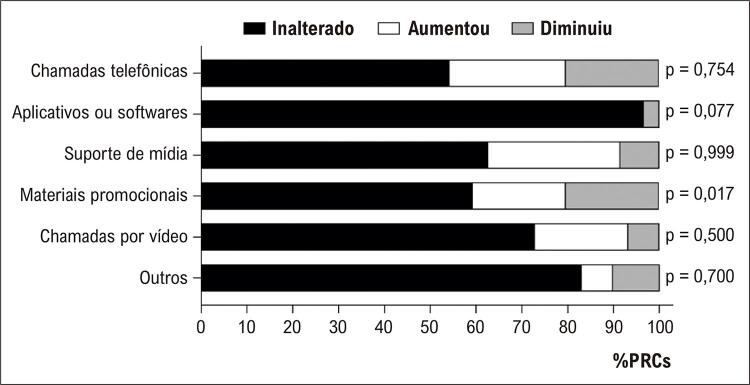
– Estratégias usadas para oferecer programas de reabilitação cardíaca remota durante a primeira onda de COVID-19 (20 a 30 de abril de 2020) nos Brasil; teste de McNemar, p<0,05; PRCs: Programas de Reabilitação Cardiovascular.

As principais adaptações feitas pelos programas que continuaram a oferecer sessões de RC durante a pandemia foram: 1) oferecer serviços remotos (n=18, 31%), 2) mudanças nas atribuições dos profissionais de saúde (n=11, 19%), 3) redução nas horas de trabalho dos profissionais de saúde (n=10, 17%) e 4) redução no

número de sessões (n=9, 15%) ( Figura 2 ). As estratégias mais utilizadas para facilitar as sessões remotas durante a pandemia foi utilizado de vídeos e fotos (aumento de 3 para 17 programas, p=0,007) e chamadas por vídeo (aumento de 3 para 12 programas, p = 0,017) ( Figura 3 ).

Somente 17 coordenadores dos PRCs relataram que os profissionais sentiam-se preparados para prover RC face as novas restrições durante o período da pandemia, e somente três PRCs (5,08%) admitiram pacientes para RC ( Figura 2 ).

A maioria dos participantes (93,22%) respondeu que o fato de receberem orientações sobre manejo da RC durante a pandemia, foi importante para um direcionamento ou orientação durante esse período. Ainda, 74,57% dos respondentes relataram necessidade de treinamento e qualificação no manejo de PRCs, e 72,88% buscaram aplicativos *online* RC remoto ( Figura 2 ).

## Discussão

Este estudo fornece informações sobre o impacto da COVID-19 nos primeiros 60 dias da pandemia (abril de 2020), destacando a redução no número de programas disponíveis durante esse crítico período. Também identificamos as principais estratégias dos PRCs para promover atividade física durante a pandemia. Segundo, apresentamos uma atualização das características demográficas, clínicas e operacionais dos PRCs no Brasil, mostrando as diferenças entre os serviços por região e reafirmando a necessidade por serviços de RC no Brasil.

### Programas de reabilitação cardiovascular antes da pandemia da COVID-19

Dos 75 PRCs no Brasil, obtivemos informações atualizadas sobre características clínicas e demográficas antes da pandemia da COVID-19 de 17 programas, e sobre o impacto de 59 programas, consistindo no maior número de PRCs avaliados por uma pesquisa no Brasil.

Assim como relatado por Britto et al.,^17^ não obtivemos nenhum respondente da região N, o que nos impediu de caracterizar os PRCs desta região. No mês anterior à pesquisa (março de 2020), houve 4358 admissões hospitalares relacionadas às doenças cardiovasculares na região N,^18^ o que reforça uma possível precariedade nessa região.

Considerando o número de pacientes hospitalizados por doenças cardiovasculares em todas as regiões brasileiras no mesmo período, e a duração média de 36 sessões para cada participante do PRC, encontramos taxas de participação extremamente baixas, variando de 1,12% na região S a 3,24% na região CO. Esses números não incluem a demanda anterior por PRCs de participantes não hospitalizados com doenças cardiovasculares, o que devia ser ainda maior, já que a taxa de hospitalização por essas condições é estimada em 11,2% de acordo com uma recente revisão sistemática.^22^ A grande demanda por PRCs, combinada com o baixo número de PRCs disponíveis, torna clara a necessidade de investimentos nessa área.

Em relação a doenças cardiovasculares, a IC foi a condição mais frequentemente relatada. No entanto, é possível que algumas doenças sejam menos mencionadas em PRCs dada sua alta complexidade, tais como o pós-transplante. Na região CO, por exemplo, pacientes no pós-transplante provavelmente não sejam encaminhados a PRCs, e a ausência de cuidado a esses pacientes nessa região também pode estar relacionada ao pequeno número de serviços públicos disponíveis. Outra possibilidade é que nem todos os programas na região CO tenham respondido o questionário. De qualquer maneira, políticas públicas para aumentar o encaminhamento de pacientes e expandir o número de PRCs na região CO são de extrema prioridade.

Quanto às características operacionais dos PRCs, nosso estudo identificou que o único componente presente em 100% dos PRCs foi a prática de exercício físico, o que foi comparável a outros estudos.^23^ Esse é um achado preocupante, uma vez que espera-se que os PRCs ofereçam todos os oito componentes essenciais: 1) exercício físico, 2) aconselhamento nutricional, 3) controle de fator de risco, 4) cessação do tabagismo, 5) suporte psicológico, 6) aconselhamento de exercício físico 7) orientação vocacional e 8) controle e adesão ao tratamento.^11^ Um estudo prévio canadense demonstrou que o aconselhamento nutricional e a orientação quanto a prática de atividade física são oferecidos por todos os PRCs.^24^ Outras inciativas públicas e privadas, com foco na cobertura nacional dos serviços e treinamento profissional são necessárias para fornecer todos os componentes necessários e, assim, otimizar a assistência a pacientes com doença cardiovascular no Brasil.

### Impactos da COVID-19 sobre programas de reabilitação cardiovascular

A primeira estratégia descrita para manter os participantes nos PRCs foi a introdução de atividades remotas, principalmente chamadas por vídeo (33%), seguido de utilização de fotos e vídeos (27%). Um estudo prévio apresentou resultados favoráveis quanto a prescrição de exercícios, adesão à reabilitação, e mudanças de comportamento pelo uso de imagens e vídeos.^25^ Outros estudos também apontaram a reabilitação remota como uma importante estratégia para aumentar a disponibilidade de RC para pacientes com doença coronariana.^26^ Esperamos que esses recursos continuem a ser incorporados mesmo após a pandemia, já que aumentar a adesão e a cobertura da RC, bem como prover mais componentes centrais a um maior número de pacientes é altamente recomendado na literatura.^27 - 29^

As atividades remotas têm sido oferecidas por diferentes abordagens. Aquelas que utilizam plataformas digitais (aplicativos ou softwares) e incluem a capacidade de monitoramento em tempo real parecem ser mais efetivas em aumentar a adesão do paciente e seu envolvimento em PRCs.^30^ Em nosso estudo, somente dois PRCs utilizaram plataformas digitais durante a pandemia da COVID-19, reforçando a necessidade urgente de melhorar os investimentos e o conhecimento nessa área, e aumentar a disponibilidade de recursos para pacientes no Brasil e provavelmente em muitos países.

Quanto ao impacto da pandemia da COVID-19 sobre os profissionais dos PRCs, encontramos que a maioria sentiam-se despreparados para os desafios apresentados pelas restrições impostas pelas COVID-19, e inseguros em prover RC durante a pandemia. Os profissionais da saúde relataram com frequência medo de serem infectados ou de infectar seus familiares, e relataram estresse emocional por mudanças nos protocolos e uso de equipamentos de proteção.^31^ Assim, é evidente que a pandemia da COVID-19 também tenha causado um impacto negativo nas rotinas dos profissionais da saúde, provavelmente interferindo na oferta, na administração, e na disponibilidade dos PRCs.

### Limitações

Este estudo teve algumas limitações, incluindo o fato de que nem todos os PRCs no Brasil tenham recebido a pesquisa *online* , que todas as questões deveriam ter sido respondidas para que a pesquisa fosse incluída no estudo, que uma ou mais questões da pesquisa possam ter sido mal interpretadas, e que a taxa de infecção por COVID-19 possa ter sido subestimada.

Além disso, a natureza transversal deste estudo não nos permite estabelecer nenhuma inferência causal. Ainda, embora tivéssemos o apoio de várias associações científicas do Brasil para assegurar uma distribuição ideal da pesquisa *online* , considerando o método de amostragem não probabilística, é possível que nem todos os PRCs no Brasil tenha recebido a pesquisa via *Internet* e, consequentemente, tenham sido sub-representados. No entanto, nosso estudo teve a maior amostra de PRCs do Brasil, fornecendo dados importantes de períodos anteriores e posteriores à pandemia da COVID-19 que ajudam a guiar PRCs no futuro. Contudo, é necessário um registro nacional e multiprofissional para facilitar a comunicação e compartilhar experiências entre os PRCs no país.

A interpretação de estudos baseados na *Internet* pode ser desafiadora. Embora as questões tenham sido desenvolvidas e cuidadosamente testadas por profissionais com ampla experiência na área de RC e manejo de PRCs, uma ou mais questões da pesquisa pode ter sido mal interpretada.

Os participantes foram solicitados a responder cada questão para prosseguirem para seções subsequentes da pesquisa online; caso contrário, não era possível acessar o formulário inteiro. Ainda, o participante podia levar o tempo que julgasse necessário para responder cada questão, uma vez que nosso formulário não requeria um tempo máximo ou mínimo para isso.

Finalmente, em relação à possível subestimação das taxas de COVID-19, a ausência de suprimentos no início da pandemia no Brasil pode ter contribuído para uma menor notificação de casos, especialmente em regiões de baixa renda. No entanto, nós utilizamos dados de entidades oficiais brasileiras, e essa condição ocorreu em outras partes do mundo no início da pandemia.

## Conclusão

Apesar da necessidade de PRCs no Brasil, o número de programas continua significativamente baixo em vista da demanda nacional e local. A pandemia da COVID-19 teve um efeito desfavorável sobre os PRCs, diminuindo o número de sessões de RC disponíveis no país, apesar de encorajar atividades de telerreabilitação durante os dois primeiros meses da pandemia da COVID-19. Uma porcentagem substancial de profissionais da saúde atuantes em PRCs não consideraram seguro realizar RC em pacientes com COVID-19 e não estavam preparados para supervisionar os programas no local. Estudos nacionais e multicêntricos analisando PRCs em diferentes estágios da pandemia da COVID-19 são necessários para avaliar seu impacto e compartilhar estratégias de sucesso a fim de mitigar os impactos negativos da pandemia sobre esses programas em todo o mundo.
