# Supplementary material for: Impacto da COVID-19 nos Programas de Reabilitação Cardiovascular no Brasil: Um Estudo Transversal Baseado em uma Pesquisa Online
Source: Arq Bras Cardiol. 2023 Feb 23;120(3):e20220135. [Article in Portuguese] doi: 10.36660/abc.20220135 (PMC9973047; doi:10.36660/abc.20220135)
Supplement: Translation

# Translation: Impact of COVID-19’s on Cardiovascular Rehabilitation Programs in Brazil: An Online Survey-Based Cross-Sectional Study

**Authors:** Iara de Sousa Cezário Jardim, Mauricio Milani, Isac Castro, Dominique Hansen, Marlus Karsten, Lawrence Patrick Cahalin, Graziella França Bernardelli Cipriano, Gerson Cipriano

**Affiliations:** 1 Brasil Programa de Pós Graduação em Ciências da Reabilitação (PPGCR), Universidade de Brasília (UNB), Brasília, DF – Brasil; 2 Brasil Fitcordis, Brasília, DF – Brasil; 3 Brasil Programa de Pós Graduação em Ciências e Tecnologias em Saúde (PPGCTS), Universidade de Brasília (UNB), Brasília, DF – Brasil; 4 Brasil Universidade de São Paulo, São Paulo, SP – Brasil; 5 Bélgica Hasselt University, Hasselt – Bélgica; 6 Brasil Programa de Pós-graduação em Fisioterapia (PPGFT), Universidade do Estado de Santa Catarina, Florianópolis, SC – Brasil; 7 EUA Miller School of Medicine, Miami, Flórida – EUA

**Keywords:** COVID-19, Telerehabilitation, Research, Surveys and Questionnaires

## Abstract

**Background:**

The COVID-19 pandemic had an impact on cardiovascular rehabilitation (CR) programs in Brazil.

**Objectives:**

To describe the characteristics of CR programs in Brazil, the impacts of the first epidemiological wave of COVID-19 (first 60 days) on the programs and present the initiatives used to overcome the impacts.

**Method:**

This cross-sectional and retrospective study utilized a specific online survey. Participants were coordinators of CR programs. Variables were presented by Brazilian geographic region and as the following categories: demographic, clinical and operational characteristics. The significance level for statistical analysis was set at 5%.

**Results:**

Fifty-nine CR programs were responsible for 5,349 patients, of which only 1,817 were post-acute cardiovascular events, which corresponded to 1.99% of hospitalized patients in the month prior to the survey (n=91,231). The greatest impact was the suspension of on-site activities, which occurred similarly in areas with the highest and the lowest rates of COVID-19 in the period. Forty-five programs (75%) were shortly interrupted, while three (5%) were ended. All 42 programs that already used remote rehabilitation strategies noticed a substantial increase in activities, based primarily on the use of media and video calling. Only three (5%) considered safe to see patients during the first 60 days.

**Conclusions:**

There was a reduction in the number of CR programs with the COVID-19 pandemic. Telerehabilitation activities increased during the first two months of the COVID-19 pandemic, but this was not enough to overcome the reduction in CR program activities across Brazil.

## Introduction

On March 11, 2020, a worldwide pandemic was declared due to the emergence of COVID-19 (abbreviation for CoronaVirus Disease 2019).^1^ There were no specific pharmaceutical products or regulated vaccines to treat the disease caused by SARS-CoV-2 during the first half of 2020.^2^ Preventative measures to reduce the rapid spread of this respiratory virus, including social distancing, were therefore recommended worldwide.^3^ The recommendation by health agencies to reduce physical contact increased the restrictions to many public and private areas, including healthcare services.^4^ This phenomenon also occurred in Brazil, where the first case was reported on February 25, 2020, nine weeks after the first reported case in China.^5^ In Brazil’s capital, Brasília, for example, the government issued its first decree for the closure of several establishments on March 11, 2020 (in the 11th epidemiological week),^6^ and these recommendations continued to change throughout the year with alterations of the restrictions still being announced in the first months of 2021.^7^

According to recent publications, chronic conditions, including pulmonary and cardiovascular diseases, diabetes mellitus, and hypertension, have been associated with more severe manifestations of COVID-19 infection and a poorer prognosis.^8 , 9^ Therefore, individuals with these conditions have been called to increase precautionary procedures and keep their clinical treatments updated, thus avoiding contamination, decompensation, and further hospitalizations.^10^

Despite being considered a vital component for heart failure treatment,^11 , 12^ the availability and adherence to cardiovascular rehabilitation (CR) were also highly affected due to new restrictions during the pandemic.^13^ CR is composed of specific core components that aim to stimulate healthy habits, promote an active lifestyle, optimize clinical conditions, and reduce cardiovascular risks,^11^ as supported by robust evidence of preventing hospitalizations and cost-effectively increasing cardiorespiratory fitness.^14 , 15^

This study aims to describe the characteristics of cardiac rehabilitation programs (CRPs) in Brazil, in its main geographic regions, before and during the pandemic, verify the impact of the COVID-19 pandemic (60 days after the first confirmed case of COVID-19 in Brazil) and report the main initiatives created to maintain the CRPs in the face of restrictions imposed by public officials.

## Methods

### Ethical approval and methodological design

In this cross-sectional and retrospective study, a customized online survey developed by CR healthcare professionals and researchers was used. The ethics committee approved the study (CAAE number 36041220.4.0000.8093). The participants signed a non-disclosure agreement on the scientific purpose and anonymous presentation of their data at an online international scientific meeting – the International Council of Cardiovascular Prevention and Rehabilitation–^16^ and further publication.

### Online survey

The online survey was developed by two researchers, to present, for the first time in the literature, Brazilian data on the impact of COVID-19 on CR, at an international scientific webinar organized by the International Council of Cardiovascular Prevention and Rehabilitation (ICCPR).^16^ The complete data collected were recorded for further assessment after approval by the ethics committee.

A free survey software (Google forms, Google LLC, California, United States) was used to develop both objective and subjective questions of the questionnaire. The questionnaire was distributed between April 20^th^and 30^th^of 2020 (60 days after the first confirmed case of COVID-19 in Brazil). The order of the questions was first aimed at characterizing CR services before the COVID-19 pandemic and, secondly, to present necessary and/or potential adaptations for the novel pandemic scenario. Although there was no time limit for respondents to complete the questionnaire, the approximate time for completing the form was set to be 10 to 15 minutes per participant. Each page had to be answered to fill the questionnaire. The Portuguese version used is still available *online* for consultation (https://forms.gle/hWiKojuAz68FpUeD6)

CR professionals were also required to agree to the terms of disclosure, consenting for the anonymous presentation of data at the ICCPR webinar and future scientific publications after ethics approval.

### Participants, and inclusion and exclusion criteria

Study participants were coordinators of CRP selected by non-probabilistic sampling. The link to access the online survey was made available via social networks, and recipients were encouraged to share the invitation with other professionals to reach as many professionals as possible (i.e., “snowball” strategy).

As inclusion criteria, the CRP needed to offer at least one of the eight core components recommended in the literature.^11^ Duplicate or partially answered questionnaires were excluded from the study.

### Statistical analysis

The Kolmogorov-Smirnov test verified the adherence of the sample data to the normal distribution. As there was no adherence, the continuous variables were described as median and interquartile range. Categorical data were presented as absolute frequency and percentages. Data were analyzed using the McNemar´s test in a 2x2 table, using: 1) the strategy promoted behavior change; 2) started or stopped using the strategy at two different points – before and after the pandemic period. Categorical data were compared using the X^2^test. The Kruskal Wallis test was used to compare medians of non-parametric data. We used a p<0.05, and a beta error ≤ 0.2 for statistical significance. Statistical analyses were performed using SPSS version 23.0 for MacOS.

A total of 57 CRPs was required in the study (76% of participants), which was calculated using a z score of 1.96 and a margin of error of 6% and considering an estimated 75 programs in Brazil.^17^ Calculations were done using the StatCal Software, EPIinfo, and the Centers for Disease Control and Prevention (CDC)’s for population questionnaires. To estimate the demand for CRP in Brazil and in each of its geographic region, we considered the number of hospitalizations for cardiovascular disease in the month prior to the survey – March 2020,^18^ (since the number of hospitalizations was similar to the previous 12-month average), divided by the number of CR sessions potentially available for the patients who had a recent acute cardiovascular event (<12 weeks).

To calculate the average rate of infection of COVID-19 for April 2020 by geographic region, we considered the number of cases registered in the national health system in 2020,^19^ divided by the number of inhabitants registered by the Brazilian Institute of Geography and Statistics for the same period.^20^ This ratio was expressed per 100,000 inhabitants.

## Results

### Characteristics of cardiovascular rehabilitation programs before the COVID-19 pandemic

The response rate to our questionnaire was 78.66% of the expected number (61 out of 75 programs). Two programs were excluded due to duplicity, resulting in 59 surveys for analysis.

The characteristics of CRPs before the COVID-19 pandemic were categorized according to the geographic regions of Brazil: north (NO), northeast (NE), mid-west (MW), southeast (SE), and south (SO) ( Table 1 ).

**Table 1:** – Demographic and clinical characteristics of cardiovascular rehabilitation programs before the first confirmed case of COVID-19 in Brazil and hospitalizations for cardiovascular diseases by Brazilian region

Demographic characteristics		Brazilian region					Total	p

		Midwest	Northeast	South	Southeast	North		
Profile of Service Provided in median (IQR) or n *(%)*	Time of Existence (month)	77 (42 -131)	68 (28 - 96)	57 (20 - 229)	141 (70 - 238)	-	-	0.26
	No. of programs	9 (15.25)	8 (13.55)	12 (20.33)	30 (50.84)	-	59	-
	Human Development Index^32^	0.757	0.663	0.754	0.766	0.667	-	-
	No. of patients per month (per program)	50 (20 - 50)	18 (7 - 65)	25 (7 - 115)	78 (49 - 180)	-	5,349	0.01
	No. of patients per month	428 (8)	270 (5.04)	985 (18.41)	3.711 (69.37)	-		
	No. of sessions per month (per program)	200 (12 - 280)	76 (12 - 220)	40 (10 - 132)	180 (50 - 400)	-	32,449	0.25
	No. of sessions per month	3,016 (9.29)	1,764 (5.43)	5,080 (15.65)	22,589 (69.61)	-		
	Percentage of Low-risk patients* per month (per program)	30 (20 - 50)	15 (5 - 55)	30 (10 - 50)	25 (20 - 50)	-	1,407	0.54
	Percentage of Moderate risk patients* per month (per program)	50 (30 - 60)	50 (20 - 65)	35 (20 - 50)	35(20 - 5)	-	2,145	0.27
	Percentage of High-risk patients* per month (per program) *	20 (10 - 20)	30 (10 - 50)	25 (5 - 55)	30 (20 - 50)	-	2,097	0.31
	Percentage of post-acute cardiovascular event patients (<12 weeks) per month (per program)	20 (20 - 30)	15 (0 - 25)	10 (5 - 30)	20 (20 - 30)	-	1,817	0.30
	No. of post-acute cardiovascular event patients (<12 weeks) per month	220 (12.1)	557 (30.7)	230 (12.7)	810 (44.6)	-		
	CRP patients covered by the public health system (per program)	0 (0 - 0)	5 (0 - 45)	25 (0 - 25)	70 (0 - 70)	-	1,173	0.48
Hospitalizations due to CVD in March 2020^29^		6,778 (7.4)	20,127 (22)	20,395 (22.3)	39,573 (43.3)	4,358 (4.7)	91,231	-
The ratio of post-acute patients in rehabilitation/Hospitalized due to CVD in March 2020 (%)		3.24	2.76	1.12	2.04	0	1.99	-
**Clinical characteristics**		**Brazilian region**					**Total**	**p**

		**Midwest**	**Northeast**	**South**	**Southeast**	**North**		
Number (%) of programs directed to specific clinical conditions	Heart failure	8 (88.9)	8 (100)	9 (75)	30 (100)	0	55 (98)	0.02
	Coronary revascularization	8 (88.9)	7 (87.5)	9 (75)	29 (96.7)	0	53 (89)	0.21
	After pacemaker implant and/or ICD	6 (66.7)	6 (75)	7 (58.3)	25 (83.3)	0	44 (74)	0.36
	Stable coronaropathy	6 (66.7)	5 (62.5)	6 (500	25 (83.3)	0	42 (71)	0.16
	Acute coronary syndrome	7 (77.8)	5 (62.5)	7 (58.3)	22 (73.3)	0	41 (69)	0.70
	Metabolic syndrome	7 (77.8)	5 (62.5)	6 (50)	23 (76.7)	0	41 (69)	0.34
	Diabetes	6 (66.7)	4 (50)	6 (50)	23 (76.7)	0	39 (66)	0.28
	Post-valve surgery	5 (55.6)	4 (50)	5 (41.7)	21 (70)	0	35 (59)	0.34
	POAD	2 (22.2)	3 (37.5)	6 (50)	18 (60)	0	30 (50)	0.21
	Adult congenital cardiac disease	3 (33.3)	2 (25)	0 (0)	11 (36.7)	0	16 (27)	0.11
	Post-cardiac Transplantation or VAD	0 (0)	2 (25)	1 (8.3)	13 (43.3)	0	16 (27)	0.02
	Others	1 (11.1)	3 (37.5)	2 (16.7)	7 (23.3)	0	13 (22)	0.51
	Post cerebrovascular event	1 (11.1)	1 (12.5)	1 (8.3)	9 (30)	0	12 (20)	0.31
	Preoperative for cardiac surgery	0 (0)	0(0)	1 (8.3)	2 (6.7)	0	3 (5)	0.72

Data expressed as an absolute number (n) and percentage (%) for categorical variables as well as the median and interquartile range (median 25-75%) for continuous variables. The comparison between groups was analyzed by X^*2*^ of Pearson (p< 0.05). IQR: interquartile range; No: number; *: Low, moderate, and high-risk patient as established in the Brazilian guideline^*26*^ ; CRP: Cardiac Rehabilitation Program; CVD: Cardiovascular Disease; ICD: Implanted Cardioverter-defibrillator; POAD: Peripheral Obstructive Arterial Disease; VAD: Ventricular Assist Device; p-value: Pearson’s chi-squared test.

Most participants (n=44, 74%) lived in four Brazilian states, which had the highest national human development index (HDI) (above 0.731), according to the literature.^21^ Considering Brazil’s average COVID-19 infection rates for the examined period (28.3 per 100,000 inhabitants), three of these four states were above the calculated number ( Figure 1 ). According to data available in the literature,^21^ the NO region had the second worst HDI in Brazil (0.667) and was the only region where there was no respondent to the questionnaire. Conversely, the SE region had the highest HDI in the country (0.766),^21^ the highest concentration of CRPs (30/59), the oldest program in Brazil (238 months), highest rate of patients treated per program (78%), and the largest number of patients covered by the public healthcare system (70%).

**Figure 1:** – Number of cardiovascular rehabilitation programs and Covid-19 infection rate before and after the first COVID-19 wave (April 20 to 30, 2020) and Human Development Index (HDI) according to federative units and regions of Brazil.

According to the number of hospitalizations for cardiovascular diseases in Brazil in March 2020 (n=91,231) and the availability of programs for patients after acute cardiovascular events (n=1,817), only 1.99% of patients could be treated in Brazilian cardiovascular rehabilitation programs. The worst scenario occurred in the SO region (number of program/number of patients: 230/20,395: 1.12%), followed by the SE region (810/39,573: 2.04%) ( Table 1 ).

As for clinical characteristics, heart failure (HF) was the most described cardiovascular clinical condition by CRPs’ coordinators (n=55, 93.2%), followed by coronary revascularization (n=53, 89.8%) and patients with a pacemaker or implantable cardioverter-defibrillator (n=44, 74.5%) ( Table 1 ). However, the percentage of programs directed at HF patients differed according to Brazil’s geographic regions. While 100% of the CRPs in the NE and in the SE directed at HF patients, only 89% and 75% of the programs in the MW and in the SO, respectively, directed at this type of patients (p=0.02) ( Table 1 ). None of the programs in the MW region directed towards post-heart transplant (post-TX) patients and/or ventricular support users; on the other hand, 43.3% of the CRPs in the SE region attended post-Tx patients (p=0.02) ( Table 1 ).

We found significant discrepancies regarding the core components employed by CRPs among the regions. Nutritional counseling was offered in one program (11%) in the MW region, and in 19 programs (63.3 %) in the SE region (p=0.005) ( Table 2 ). Vocational counseling was the least frequently offered component in Brazil (n=1, 8.3%), while exercise (n=59, 100%) and physical activity (n=57, 96%) counseling were the most frequent ones ( Table 2 ).

**Table 2:** – Operational characteristics of cardiovascular rehabilitation programs before the first confirmed case of COVID-19 in Brazil and hospitalizations for cardiovascular diseases by Brazilian region

Operational characteristics		Brazilian region						Total	p

		Midwest	Northeast	South		Southeast	North		
Number (%) of programs that offered specific core components	Exercises	9 (100)	8 (100)	30 (100)		12 (100)	0	59 (100)	1
	Nutritional counseling	1 (11.1)	6 (75)	10 (83.3)		19 (63.3)	0	36 (61)	0.005
	Control of risk factors	7 (77.8)	7 (87.5)	9 (75)		26 (86.7)	0	49 (83)	0.07
	Quitting tobacco use	1 (11.1)	2 (25)	3 (25)		9 (30)	0	15 (25)	0.72
	Psychological support	1 (11.1)	3 (37.5)	5 (41.7)		12 (40)	0	21(35)	0.42
	Counseling on physical activity	8 (88.9)	8 (100)	11(91.7)		30 (100)	0	57 (96)	0.27
	Vocational support	0 (0)	0 (0)	1 (8.3)		0 (0)	0	1 (1)	0.80
	Treatment adhesion control	7 (77.8)	7 (87.5)	8 (66.7)		25 (83.3)	0	47 (79)	0.61
Assessment methods used by the programs *n (%)*	Cardiopulmonary exercise test	4 (44.4)	7 (87.5)	6 (50)		20 (66.7)	0	37 (62)	0.22
	6-Minute walk test	7 (77.8)	4 (50)	11(91.7)		17 (56.7)	0	39 (66)	0.10
	Sit-to-Stand test	3 (33.3)	4 (50)	7 (58.3)		12 (40)	0	26 (44)	0.36
	Handgrip strength test	2 (22.2)	3 (37.5)	6 (50)		9 (30)	0	20 (33)	0.53
	Respiratory muscle strength	4 (44.4)	6 (75)	7 (58.3)		17 (56.7)	0	34 (57)	0.65
	Flexibility test	3 (33.3)	3 (37.5)	4 (33.3)		14 (46.7)	0	24 (40)	0.81
	Functional mobility – Timed up and go test	2 (22.2)	1(12.52)	4 (33.3)		8 (26.7)	0	15 (25)	0.75
	Balance evaluation – Berg's Test	1 (11.1)	2 (25)	1 (8.3)		9 (30)	0	13 (22)	0.37
	Quality of life test	1 (11.1)	2 (25)	1 (8.3)		2 (6.7)	0	6 (10)	0.49
Types of Training offered by the programs *n (%)*	Motor coordination training	4 (44.4)	5 (62.5)	6 (50)		25 (83.3)	0	40 (68)	0.06
	Balance training	5 (55.6)	6 (75)	8 (66.7)		27 (90)	0	46 (78)	0.10
	Stretching	8 (88.9)	8 (100)	12 (100)		30 (100)	0	58 (98)	0.13
	Moderate-intensity respiratory muscle training	2 (22.2)	6 (75)	6 (50)		17 (56.7)	0	31 (53)	0.16
	Moderate-intensity resistance training	7 (77.8)	7 (87.5)	11 (91.7)		28 (93.3)	0	53 (90)	0.58
	High-intensity interval training	5 (55.6)	6 (75)	4 (33.3)		17 (56.7)	0	32 (54)	0.31
	Moderate-intensity continuous training	8 (88.9)	8 (100)	11 (91.7)		30 (100)	0	57 (97)	0.27
Strategies used to offer remote cardiac rehabilitation *n (%)*	Phone calls	2 (22.2)	3 (37.5)	4 (33.3)		11 (36.7)	0	20 (33)	0.87
	App or software for CRP	0 (0)	0 (0)	0 (0)		0 (0)	0	0 (0)	1.00
	Media support (videos and photos)	0 (0)	1 (12.5)	2 (16.7)		5 (16.7)	0	8 (13)	0.62
	Promotional material	1 (11.1)	1 (12.5)	2 (16.7)		11 (36.7)	0	16 (27)	0.24
	Video calls	0 (0)	1 (12.5)	2 (16.7)		4 (13.3)	0	7 (11)	0.67
	Others	1 (11.1)	1 (12.5)	1 (8.3)		4 (13.3)	0	7 (11)	0.97

*Data expressed as an absolute number (n) and percentage (%) for categorical variables. Comparisons between groups were analyzed by Kruskal-Wallis (p<0.05); CRP: cardiac rehabilitation program; p-value: Kruskal-Wallis test.*

The main exercise modalities offered by CRPs for in-person sessions were stretching (98%), moderate continuous aerobic training (97%), and moderate-intensity resistance training (90%) ( Table 2 ). None of the 59 programs used specific CR software or applications before the COVID-19 pandemic; telephone calls (n=20, 33%) and promotional materials (n=16, 27%) were the most prevalent remote initiatives before the pandemic period ( Table 2 ).

### Impact of COVID-19 on Brazil’s cardiovascular rehabilitation programs

According to the national average of COVID-19 infection, Brazilian states were differently affected in April 2020 (28.3 per 100,000 inhabitants). In the SE region, 23 (63.9%) of the CRPs were in cities with an infection rate above the national average, while 5.6% of the CRPs in the SO, 19.4% in MW, and 11. 1% in NE were conducted in cities with an infection rate above the national average (p=0.002) ( Table 3 ).

**Table 3:** – Place of cardiovascular rehabilitation program according to the national average rate of COVID-19 (> or ≤28.3 per 100,000 inhabitants) per Brazilian region and the main impacts of the programs in the first 60 days after the first case of COVID-19 in Brazil

Regions of Brazil/Impacts of the pandemic	National average rate of COVID-19 calculated in the 60 days of pandemic								Pearson’s chi-square test

	< = 28.3 per 100,000 inhabitants				> 28.3 per 100,000 inhabitants				

	n	%			n	%	Total (n)	Total (%)	
Midwest	2	8.7			7	19.4	9	15.2	p = 0.002
Northeast	4	17.4			4	11.1	8	13.6	
Southeast	7	30.4			23	63.9	30	50.9	
South	10	43.5			2	5.6	12	20.3	
*Total*	*23*	*100*			*36*	*100*	59	100	
Closing of the program	2	8.7			1	2.8	3	5	p = 0.09
Interruption of the program	15	65.2			30	83.3	45	76.3	
Reduction in the number of sessions	6	26.1			2	5.6	8	13.6	
Normal functioning of the program	0	0			1	2.8	1	1.7	
Others	0	0			2	5.5	2	3.4	
***Total***	*23*	*100*			*36*	*100*	59	100	

*Categorical variables expressed as absolute number (n) and percentage (%). The comparison between groups was analyzed by Pearson’s chi-square test (p< 0.05).*

The greatest impact of the COVID-19 pandemic on CRPs was the city-wide commercial restrictions, which occurred regardless of the local infection rate, as 30 of 36 (83.3%) programs were in cities with a rate above the average, and 15 of 23 (65.2%) programs were in cities with a rate below the average ( Table 3 ).

A reduction in the number of CR sessions offered was the second greatest impact, observed in 26.1% of the cities with the highest COVID-19 infection rates (26.1%) and in 5.6% in the cities with the lowest COVID-19 infection rates (p=0.097) ( Table 3 ).

The main adaptations made by the programs that continued to offer CR sessions during the pandemic were: 1) offer or remote services (n=18, 31%), 2) changes in the duties of health professionals (n=11, 19%), 3) reducing working hours of health professionals (n=10, 17%) and 4) reducing the number of sessions (n=9, 15%) ( Figure 2 ). The most used strategies to facilitate remote sessions during the COVID-19 pandemic were videos and photos (expanded from three to 17 programs, p=0.007) and video calls (expanded from three to 12 programs, p = 0.017) ( Figure 3 ).

**Figure 2:** – Most frequent observed and expected changes in cardiac rehabilitation programs (CRP) imposed by the COVID-19 pandemic and CRP professionals’ perceptions about managing COVID-19 patients during the first pandemic wave (April 20 to 30, 2020) in Brazil.

**Figure 3:** – Strategies used to offer remote cardiac rehabilitation programs during the first wave of COVID-19 (April 20 to 30, 2020) in Brazil; McNemar test was used for statistical analysis, p<0.05.

Only 17 CRPs’ coordinators (28.81%) reported that health professionals felt prepared to provide CR in the face of new restrictions during the pandemic period, and only three CRPs (5.08%) admitted patients to CR ( Figure 2 ).

Most participants (93.22%) responded that having an official statement on CR, issued by Brazilian scientific associations during the pandemic COVID-19, was important in getting a direction or orientation during this unprecedented time. Additionally, 74.57% of respondents also reported a need for training and qualification in managing CRPs, and 72.88% sought online applications on remote CR ( Figure 2 ).

## Discussion

This study provides information regarding the impact of COVID-19 in the first 60 days of pandemic (April 2020), highlighting the large reduction in the number of programs available during this critical period. We also identified the main strategies of CRPs to promote physical activity throughout the pandemic. Secondly, we presented an update on the demographic, clinical, and operational characteristics of CRPs in Brazil, showing the differences among services by region and reaffirming the need for CR services in Brazil.

### Cardiovascular rehabilitation programs before the COVID-19 pandemic

Of the estimated 75 CRPs in Brazil, we obtained updated information on demographic and clinical characteristics prior to the COVID-19 pandemic from 17 programs and the impact of 59 programs, which is the largest number of CRPs assessed by survey in Brazil.

As experienced by Britto et al.,^17^ we did not have any respondents from the NO region, preventing us from characterizing the CRPs of this region. In the month prior to the survey (March 2020), there were 4,358 hospital admissions related to cardiovascular diseases in the NO region,^18^ which further reinforces a possible precarious situation in this region.

Considering the number of patients hospitalized for cardiovascular diseases in all Brazilian regions in the same period, and the mean duration of 36 sessions for each CR patient, we found extremely low participation rates, ranging from only 1.12% in the SO region to 3.24 % in the MW region. These numbers do not include the previous demand for CRPs from non-hospitalized participants with cardiovascular diseases, which we assume to be even higher since the rate of hospitalization for these conditions is estimated at 11.2%, according to a recent systematic review.^22^ The great demand for CRPs combined with the low number of CRPs available makes it clear there is a considerable need for investments in this area.

Regarding cardiovascular diseases, HF was the most frequently reported condition. However, some diseases may be less frequently mentioned in CRPs due to their high complexity, such as post-transplant. In the MW region, for example, post-transplant patients are probably not referred to CRPs, and the lack of care for these patients in this region may also be related to the low number of public health services available. Another possibility is that not all programs in the MW region have answered the online survey. In any case, public policies to increase patient referral and expand the number of CRPs in the MW region are of extreme priority.

As for the operational characteristics of CRPs, our study identified that the only core component present in 100% of CRPs was exercise, which was similar to other studies.^23^ This is a disturbing finding since CRPs are expected to offer all eight core components: 1) physical exercise, 2) nutritional counseling, 3) risk factor control, 4) smoking cessation, 5) psychological support, 6) physical exercise counseling, 7) vocational support and 8) treatment adherence and control.^11^ A previous study in Canada demonstrated that nutritional counseling and guidance in physical activity are provided in all CRPs.^24^ Additional public and private initiatives focused on the national coverage of services and professional training seem necessary to deliver all the required core components and thus optimize the care of patients with cardiovascular disease in Brazil.

### Impacts of COVID-19 on cardiovascular rehabilitation programs

The first strategy described to keep participants in CRPs was the introduction of remote activities, mostly video calls (33%), followed by photos and videos (27%). A previous study showed favorable results for exercise prescription, adherence to rehabilitation, and behavioral changes through the usage of images and videos.^25^ Other studies also indicated remote rehabilitation as an important strategy to increase the availability of CR for patients with coronary disease.^26^ We hope that these resources will continue to be incorporated even after the pandemic, as increasing CR adherence and coverage, as well as delivering more core components for a greater number of patients is highly recommended in the literature.^27 - 29^

Remote activities have been offered by different approaches. Those that use digital platforms (applications or software) and include prescription and real-time monitoring capabilities appear to be more effective in increasing patient adherence and involvement in CRPs.^30^ In our study, only two CRPs used digital platforms during the COVID-19 pandemic, reinforcing the urgent need to improve investments and knowledge in this field while increasing the availability of resources for patients in Brazil and probably in many other nations.

Regarding the impact of the COVID-19 pandemic on CRP professionals, we found that most of them felt unprepared for the challenges presented by COVID-19 restrictions and insecure in providing CR during the pandemic. Health professionals often reported fear of becoming infected or infecting their families and reported emotional distress due to changes in protocols and the use of protective equipment.^31^ It is therefore clear that the COVID-19 pandemic also negatively impacted health professionals’ routines, likely interfering with the delivery, management, and availability of CRPs.

### Limitations

This study had some limitations, including the fact that not all CRPs in Brazil may have received the survey and all the questions must have been answered to include the survey in the study; that one or more questions on the online survey may have been misinterpreted, and that calculation of the COVID-19 infection rate may have been underestimated.

In addition, the cross-sectional nature of this study precludes any causal inference. Furthermore, although we had the support of several scientific associations in Brazil to ensure the ideal distribution of the online survey, considering the non-probabilistic sampling method utilized, it is possible that not all CRPs in Brazil received the survey via the internet, and consequently were underrepresented. However, our study had the largest sample size of CRPs in Brazil, providing important data from before and after the COVID-19 pandemic that help guide CRPs in the future. However, a national, multidisciplinary registry is needed to facilitate communication and share experiences among CRPs across the country.

Interpretation of Internet-based studies can be challenging. Although the questions had been developed and carefully tested by professionals with extensive experience in the field of CR and CRP management, one or more of the survey questions may have been misinterpreted.

The respondents were required to answer every question to be able to continue to subsequent sections of the online survey; otherwise, it was not possible to access the entire form. Also, the respondent could take the time necessary to answer each question, as our form did not require a minimum or maximum time for that.

Finally, regarding the possible underestimation of COVID-19 infection rates, the lack of supplies at the beginning of the pandemic in Brazil may have contributed to a lower notification of cases, especially in low-income regions. However, we used data from official Brazilian entities, and this condition occurred in other parts of the world at the beginning of the pandemic.

## Conclusion

Despite the substantial need for CRPs in Brazil, the number of programs remains significantly low in view of national and local demand. The COVID-19 pandemic had an unfavorable effect on CRPs by decreasing the number of CR sessions available nationwide despite encouraging telerehabilitation activities during the first two months of the COVID-19 pandemic. A substantial percentage of health professionals in CRPs did not consider it safe to perform CR with COVID-19 patients and were not prepared to supervise the programs on-site. National and multicenter studies analyzing CRPs at different stages of the COVID-19 pandemic are needed to assess their impacts and share successful strategies for mitigating the negative impacts of the pandemic on these programs worldwide.
